# Inequity in the utilization of antenatal and delivery care in Yangon region, Myanmar: a cross-sectional study

**DOI:** 10.1186/s12939-018-0778-0

**Published:** 2018-05-22

**Authors:** Aye Nyein Moe Myint, Tippawan Liabsuetrakul, Thein Thein Htay, Myint Myint Wai, Johanne Sundby, Espen Bjertness

**Affiliations:** 1 International Relations Division, Ministry of Health and Sports, Nay Pyi Taw, Myanmar; 2 Epidemiology Unit, Faculty of Medicine, Prince of Songkla University, Hat Yai, Songkhla, Thailand; 3 Ministry of Health and Sports, Nay Pyi Taw, Myanmar; 4 Department of Medical Services (Planning), Ministry of Health and Sports, Nay Pyi Taw, Myanmar; 5 Department of Community Medicine and Global Health, University of Oslo, Oslo, Norway

**Keywords:** Inequity, Wealth index, Concentration index, Antenatal care, Delivery care

## Abstract

**Background:**

Equity of access to and utilization of healthcare across socio-economic groups is important to achieve universal health coverage. Although the utilization of antenatal and delivery care has been increasing in low- and middle-income countries, inequities in the utilization of antenatal and delivery care have been reported in many countries, but have not yet been studied in Myanmar. This study aimed to determine whether inequities in the utilization of antenatal and delivery care existed in Yangon region, Myanmar.

**Methods:**

A community-based cross-sectional survey using multistage sampling was conducted from October to November 2016. A wealth index was selected as the main socioeconomic parameter for measuring inequities with respect to early initiation of antenatal care (ANC), number of antenatal care visits, delivery by a skilled birth attendant (SBA) and delivery by cesarean section (CS). Inequities were evaluated using concentration curves and concentration indexes.

**Results:**

Of the 762 women who gave birth within the 12-month survey period, there was no evidence of inequity in utilization of ANC; however, inequity of at least one antenatal visit among women aged less than 20 years was found with a concentration index of 0.04. The concentration indexes for delivery by SBA and CS were 0.05 and 0.14, respectively. Delivery by CS was disproportionately higher in adolescents and women with higher education than middle school.

**Conclusion:**

There was no overall inequity in the utilization of ANC but substantial inequities in delivery by CS and SBA were shown. Social determinants of health, particularly age and education, were associated with inequities in the utilization of delivery care. Adolescent pregnant women were found to be particularly vulnerable, and thus should be a target group for strategic plans to reduce inequities in utilization of delivery care.

## Background

Globally, 303,000 women died from the complications of pregnancy and childbirth in 2015 according to United Nations Sustainable Development Goals (SDGs) report 2017 [1]. Maternal deaths, deaths due to pregnancy and childbirth, remain the leading cause of death among women of reproductive age in low-income countries [2]. It has been shown that timely and appropriate antenatal care (ANC) and delivery by a skilled birth attendant (SBA) improve pregnancy outcomes and reduce maternal deaths [3]. According to the World Health Statistics SDGs report in 2017, maternal deaths and utilization of delivery care by SBA showed improvement in many countries [4]. However, disparities in the utilization of maternal health care services has been reported in many low- and middle-income countries, in most cases due to financial or socioeconomic barriers [5]. Similarly, the maternal and infant deaths has been decreasing and utilization of maternal health services have been improved in Myanmar as same as other Asian countries; however, these were higher than other countries such as Thailand, Singapore, and Brunei [6, 7].

Although the ability to seek and receive health care services should be equal for all socio-economic groups in the interests of fairness and social justice [8], either equity or inequity in the utilization of ANC and delivery care have manifested differently across countries [5, 9]. For example, a study in Thailand showed no inequity of accessing maternal health care [9]. In contrast, there were inequities in the utilization of maternal health care services in some countries in Asia such as Vietnam, Bangladesh, and Nepal [10–12]. Substantial socioeconomic gaps, defined by wealth and level of education, were shown to be related with maternal health care services in previous studies [5, 13]; however, the analyses of inequities are limited in Myanmar.

According to the World Bank and Myanmar Demographic and Health Survey report, socio-economic indicators such as GDP per capita, poverty rates, and the utilization of ANC and delivery care have improved in recent years, but there is no evidence concerning whether inequities in the utilization of maternal health care services is better than in the past [14–16]. Strengthening universal health coverage (UHC) to reduce the financial burden on the poor and vulnerable is emphasized in the Myanmar national health plan [17, 18]. Therefore, this study aimed to determine whether inequities in the utilization of ANC and delivery care existed in Yangon region, Myanmar. The findings can lead to better understanding of whether inequity still exists or has been alleviated in utilization of maternal health care services in Myanmar which will be essential for monitoring and evaluation of maternal health in the future.

### ANC and delivery care in Myanmar

The coverage of ANC and delivery care in Myanmar has been improved. The ANC coverage was increased from 63.1% in 2005 to 86.1% in 2016. Likewise, the delivery by SBA was increased from 64.4% in 2009 to 78.4% in 2016 [19]. In the rural areas, the ANC and delivery care can be provided in the Rural Health Centers and Sub-Rural Health Centers. For urban areas, the ANC and delivery care services are provided in Primary and Secondary Health centers and Maternal and Child Health centers. Those who need referral are referred to a Station hospital in rural areas or a Township hospital in urban areas [17]. Public and private facilities are available for ANC and delivery care services but only 10% of delivery occurred in private facilities in a Public Health Statistic report in 2016 [19].

Free ANC and delivery care services were offered in both public facility-based and primary health care settings in Myanmar [17, 20]. The national budget expenditure for Maternal and Child Health function was increased nearly three times from 2009/2010 budget year to 2013/2014 budget year [20]. Although the government policy emphasized on increasing budget investment and free of charge services, there were the reports on out-of-pocket payment for ANC and delivery care [21].

## Methods

### Study setting and design

A community-based cross-sectional survey was conducted from October 2016 to November 2016 in Yangon Region of Myanmar. Yangon region located in the lower part of Myanmar having the largest population size [22] was selected to be a study area because the coverage of ANC and delivery by SBA was 95% and 83%, respectively. However, high maternal mortality ratio of 213 per 100,000 live births was reported in 2016 [14, 23].

### Study sample, sample size and sampling methods

Women aged 15–49 years with a history of delivery within the past 12 months residing in the selected districts were included in the sample. Mentally retarded or seriously ill women were excluded. From previous studies, the rates of ANC and delivery care utilization between the richest and poorest quintiles of the wealth index were 85% vs 95% and 51% vs 96%, respectively [24]. According to higher gap difference for delivery care, we used the utilization of ANC to get the biggest samples to cover for the utilization of ANC and delivery care. The sample size was calculated based on the rate of ANC utilization between the richest and poorest quintiles of the wealth index (85% vs 95%) using the two-proportion difference formula with a 95% confidence interval and type II error of 20% [25]. According to a design effect of 2 and estimated 10% non-response rate, at least 700 women were required for the study.

A multi-stage sampling technique was used in our study. The Yangon region is divided into four districts (north, south, west and east). West and east districts are the central part of Yangon having only urban populations. Firstly, the north and south districts were selected in our study because these two districts are the main-landed areas of Yangon including both urban and rural population. Secondly, the wards and villages within the districts were taken by proportional probability sampling (PPS) considering the actual proportion of urban and rural population accounted for 50–50% in the north districts and 30–70% in the south districts. The units of urban and rural population are wards and villages, respectively. A total of 125 wards and 235 villages are in the north district and 110 wards and 375 villages are in the south district [26, 27]. Eight wards and eight villages in the north and south districts were randomly selected leading to a total 16 study wards and 16 study villages were randomly selected. Finally, the household having a woman who had delivered in the previous 12 months was randomly selected.

The household with eligible women were obtained from the immunization records which were maintained by the midwives in each ward or village which are routinely reported monthly to higher-level administrative section [28]. The immunization coverage of infants in Yangon region is more than 90% which cover infants from women delivered by SBA and traditional birth attendants [14]. The households in the selected wards and villages were visited by the research team to identify the women based on the inclusion criteria. If any household had more than one eligible woman, only one was randomly chosen for the study.

### Study variables

The main study outcome was inequities in the utilization of ANC and delivery care. Inequity considering the wealth index in relation to utilization of care was determined by evaluating the concentration curve and index. The wealth index was calculated from household characteristics and assets based on the 2014 Myanmar census report using the principal component analysis and divided into five wealth quintiles, namely poorest, second, third, fourth and richest quintile [29]. Utilization of ANC was divided into utilization of at least one-visit ANC, early initiation of ANC defined as initiation of first antenatal visit within three months of gestational age and at least four-visit ANC. Utilization of delivery care included delivery by a SBA and cesarean section. Maternal characteristics including age (age in years at the time of the survey), level of education (highest level of education at the time of survey), and number of births were independent variables.

### Data collection

#### Preparatory phase

After obtaining ethical approval, the study questionnaire was pre-tested among women aged 15–49 years with characteristics similar to the study inclusion criteria to ensure the clear meaning the variables collected in the questionnaire. The pre-test was conducted in women who were not in the study areas and the final version of questionnaire was used for data collection. A two-day workshop was held for all research assistants where they were trained in data collection before the field survey and on how to conduct a quantitative interview including checking for completeness of the information.

#### Data collection phase

The lists of targeted women were obtained and all eligible women were made appointment before the research team visited. The research team visited women’s home and invited them to participate in our study. For the women who were not available on the day the team went to visit, we returned back for their availability until three visits. The interviews with the participating women were conducted at each woman’s convenience at their home. After signing the consent forms, they were interviewed privately using the structured questionnaire by either the principal investigator or one of four trained research assistants. Data completeness was checked on a daily basis.

### Data analysis

The data were recorded in EpiData 3.1 on a double entry basis [30] and the analysis was performed using R version 3.4.2 [31]. Categorical variables are described by frequencies and percentages. The Chi-square test was used to assess the associations between level of education and wealth quintiles. Inequities in the utilization of ANC and delivery care were determined by evaluating concentration curves and concentration indexes.

The concentration curve plotted the cumulative fraction of utilization of ANC and delivery care against the cumulative fraction of women ranked by wealth quintiles [32]. The line of equality is drawn 45 degrees diagonally from the bottom left corner to the top right corner in the concentration curve. The curve lines represented each indicator of utilization of ANC and delivery care and demonstrated how far they deviated from the line of equality. The line above the equality line indicates concentration of utilization among those who are the poorer, while the line below the equality line shows concentration of utilization among those who are the richer.

In the calculation of a concentration index, the women were ranked by increasing wealth quintiles. When a concentration index equals zero, it indicates no inequity and the theoretical maximums of a concentration index range from + 1 to − 1. Negative and positive values of a concentration index show when the utilization of ANC and delivery care are concentrated among women in the poorest quintile and those in the richest quintile, respectively [33]. In our study, the concentration index of utilizing ANC and delivery care of the richest to poorest quintiles was stratified by age, level of education and number of births. A *p* value less than 0.05 was considered as statistically significant.

## Results

The response rate was 100% among the 762 women invited to participate in the study. Most participants were aged 20–34 years and 61% had a middle school or above level of education. The women were equally distributed among the wealth quintiles. Three-fourths of the women had 1–2 children. Almost all women had received ANC at least once, and 79% had had four visits or more. Early initiation of ANC was reported in only one-third of the women. Of all women, 88.5% and 26.8% of them delivered with a SBA or by cesarean section, respectively (Table 1).

**Table 1:** Characteristics of the study women (*n* = 762)

Characteristic	n (%)
Background and maternal characteristics	
Age	
< 20 years	28 (3.7)
20–34 years	565 (74.1)
≥ 35 years	169 (22.2)
Education	
No formal school	46 (6.0)
Primary school	252 (33.1)
Middle school or above	464 (60.9)
Wealth quintile	
Poorest quintile	153 (20.1)
2nd quintile	152 (19.9)
3rd quintile	152 (19.9)
4th quintile	152 (19.9)
Richest quintile	153 (20.1)
No. of births	
1–2	568 (74.5)
> 2	194 (25.5)
Antenatal care characteristics	
At least one antenatal visit	
No	21 (2.8)
Yes	741 (97.2)
Early initiation of antenatal care	
No	506 (66.4)
Yes	256 (33.6)
At least four antenatal visits	
No	162 (21.3)
Yes	600 (78.7)
Delivery care characteristics	
Delivery by a skilled birth attendant	
No	88 (11.5)
Yes	674 (88.5)
Cesarean section	
No	558 (73.2)
Yes	204 (26.8)

**Fig. 1:**
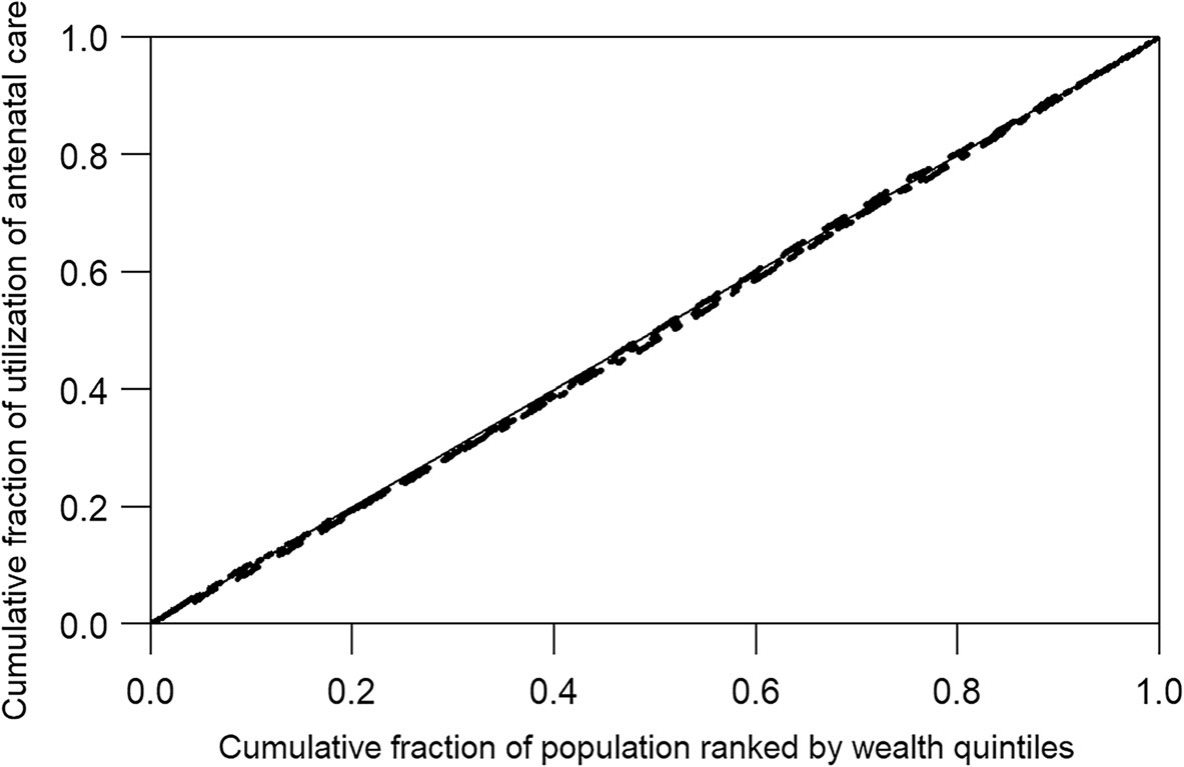
Concentration curve for utilization of ANC.  Line of equality;  At least one antenatal visit (concentration index = 0.01);  Early initiation of antenatal care (concentration index = 0.01);  At least four antenatal visits (concentration index = 0.02)

**Fig. 2:**
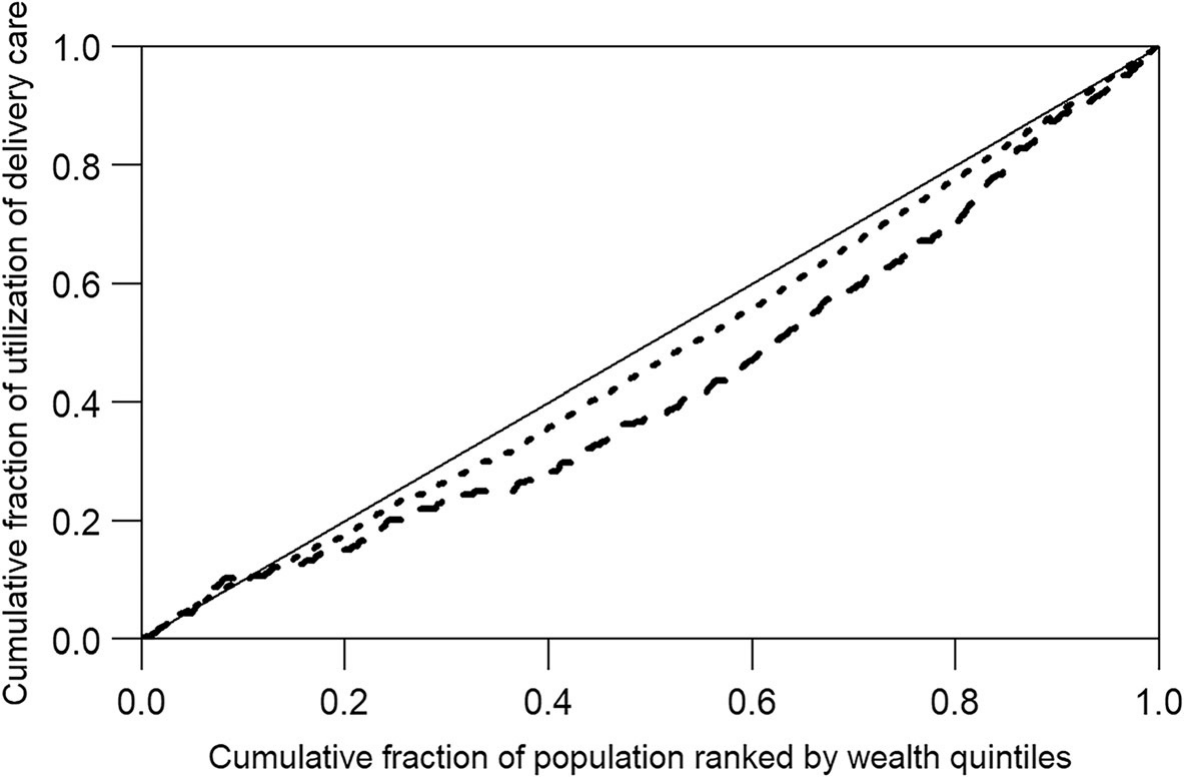
Concentration curve for utilization of delivery care;  Line of equality;  Delivery by a skilled birth attendant (concentration index = 0.05);  Delivery by cesarean section (concentration index = 0.14)

There was no evidence of inequities in utilization of ANC based on the results of the concentration curve and indexes (Fig. 1). The concentration curve was disproportionately in favor of rich women for both deliveries by a SBA and by cesarean section, with concentration indexes of 0.05 and 0.14, respectively (Fig. 2). When the inequity among different characteristics was considered, the utilization of at least one antenatal visit was more common in rich women aged less than 20 years (concentration index 0.04, 95% CI 0.001–0.079) than in the other age groups (Table 2).

**Table 2:** Inequities in the utilization of antenatal care by different maternal characteristics

	Q1 (%) Poorest	Q2 (%)	Q3 (%)	Q4 (%)	Q5 (%) richest	Concentration Index (95% CI)
At least one antenatal visit						
Age						
< 20 years	2.8	5.6	4.0	3.3	2.0	0.04 (0.001,0.079)*
20–34 years	71.7	72.2	73.8	75.3	78.4	0.01 (0.002,0.018)*
≥ 35 years	25.5	22.2	22.2	21.4	19.6	0.01 (-0.002,0.022)
Education						
No formal school	12.4	8.3	4.0	2.7	1.3	0.02 (0.001,0.040)*
Primary school	57.2	43.1	28.2	17.3	19.0	0.01 (-0.002,0.023)
Middle school or above	30.4	48.6	67.8	80.0	79.7	0.01 (-0.002,0.010)
No. of births						
1–2	62.1	72.9	71.1	82.7	85.0	0.01 (0.006,0.014)*
> 2	37.9	27.1	28.9	17.3	15.0	0.01 (0.001,0.020)*
Early initiation of antenatal care						
Age						
< 20 years	3.1	13.9	0.0	4.2	0.0	0.07 (−0.087,0.227)
20–34 years	78.1	60.5	78.3	83.0	76.8	0.00 (−0.074,0.082)
≥ 35 years	18.8	25.6	21.7	12.8	23.2	0.00 (− 0.080,0.076)
Education						
No formal school	9.4	11.6	2.2	4.3	1.8	0.00 (− 0.075,0.081)
Primary school	60.9	41.9	32.6	23.4	12.5	0.02 (−0.039,0.079)
Middle school and above	29.7	46.5	65.2	72.3	85.7	−0.01 (− 0.069,0.049)
No. of births						
1–2	68.8	76.7	76.1	89.4	92.9	0.00 (−0.057,0.061)
> 2	31.2	23.3	23.9	10.6	7.1	0.02 (0.001,0.040)*
At least four antenatal visits						
Age						
< 20 years	1.8	6.7	4.0	3.2	2.3	0.04 (−0.019,0.099)
20–34 years	74.3	73.3	72.8	76.6	78.2	0.01 (-0.004,0.024)
≥ 35 years	23.9	20.0	23.2	20.2	19.5	0.02 (−0.019,0.059)
Education						
No formal school	13.3	7.6	1.6	2.4	0.8	−0.04 (−0.138,0.058)
Primary school	55.7	39.1	25.6	24.2	18.0	0.01 (−0.029,0.049)
Middle school or above	31.0	53.3	72.8	81.4	81.2	0.01 (−0.009,0.030)
No. of births						
1–2	64.6	74.3	74.4	87.9	85.0	0.02 (0.001,0.040)*
> 2	35.4	25.7	25.6	12.1	15.0	0.00 (−0.057,0.061)

*Q* Quintile

*CI* Confidence Interval

*p* < 0.05 *, *p* < 0.01 **, *p* < 0.001 ***

**Fig. 3:**
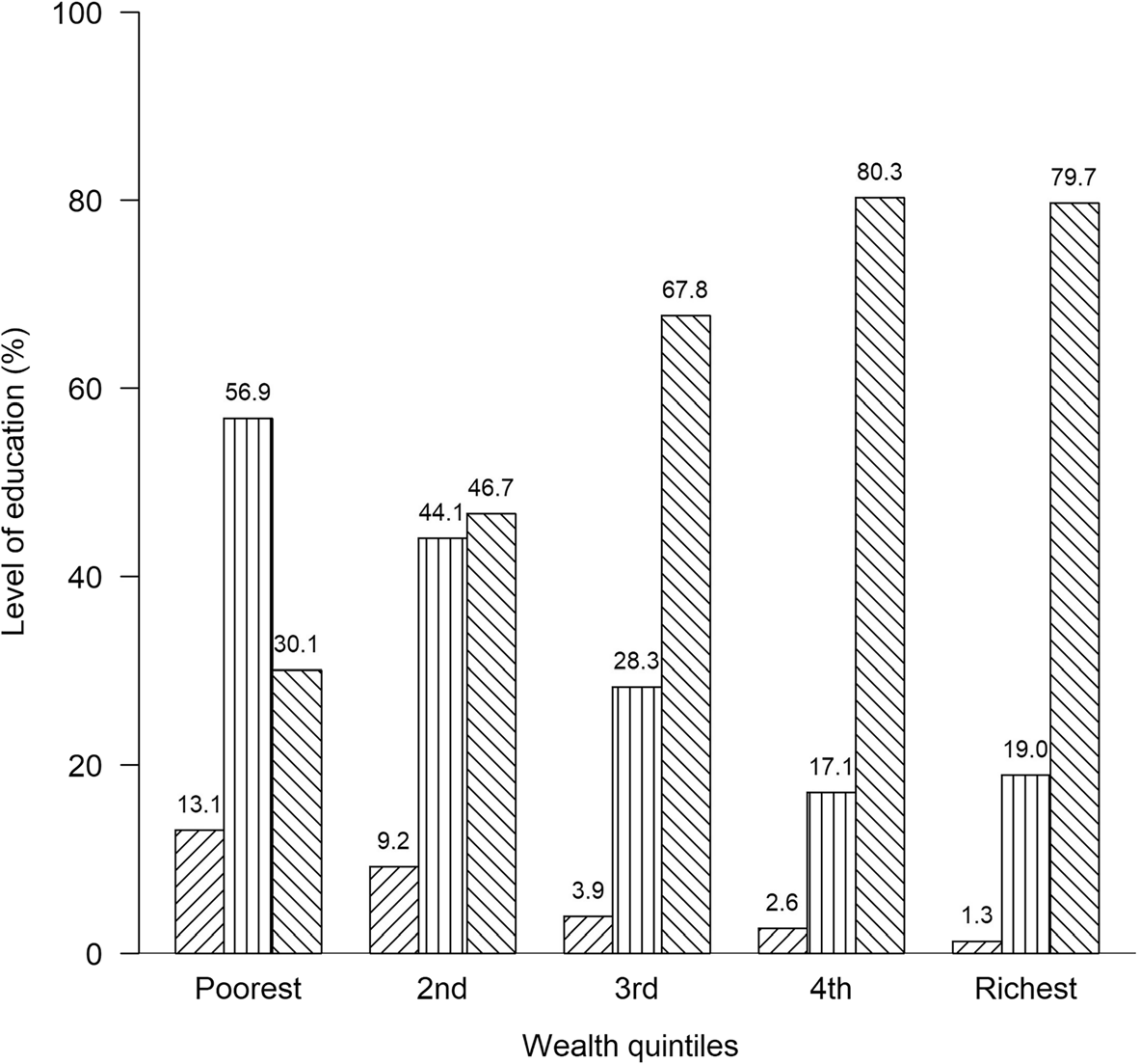
Relationship between level of education and wealth quintiles;  No education;  Primary school;  Middle school and above; *p* < 0.001

Table 3 shows the inequity in the utilization of delivery care by different maternal characteristics. Delivery by a SBA among women was disproportionately concentrated among the rich women regardless of maternal characteristics. Delivery by cesarean section was more commonly found among adolescent women and those with a middle school or above who were rich. Education-related inequities for utilization of ANC and delivery care were similar to wealth-related inequities. Figure 3 shows the positive association between level of education and wealth quintiles, and it can be seen that the higher the level of education, the richer the population, and the lower the level of education, the poorer the population.

**Table 3:** Inequities in the utilization of delivery care by different maternal characteristics

	Q1 (%) Poorest	Q2 (%)	Q3 (%)	Q4 (%)	Q5 (%) richest	Concentration Index (95% CI)
Delivery by a skilled birth attendant						
Age						
< 20 years	3.3	5.8	3.0	3.4	2.0	0.06 (−0.038,0.158)
20–34 years	71.7	75.2	73.9	76.3	78.1	0.04 (0.020,0.060)***
≥ 35 years	25.0	19.0	23.1	20.3	19.9	0.06 (0.021,0.099)**
Education						
No formal school	10.8	7.5	2.2	2.7	1.3	0.02 (−0.98,0.138)
Primary school	56.7	38.8	25.4	16.9	17.9	0.03 (−0.009,0.069)
Middle school or above	32.5	53.7	72.4	80.4	80.8	0.02 (0.001,0.040)*
No. of births						
1–2	65.8	75.2	71.6	83.1	84.8	0.04 (0.030,0.050)***
> 2	34.2	24.8	28.4	16.9	15.2	0.07 (0.050,0.090)***
Delivery by Cesarean section						
Age						
< 20 years	3.2	0.0	0.0	2.1	1.6	0.25 (0.211,0.289)***
20–34 years	64.5	69.2	71.8	70.2	67.2	0.13 (0.071,0.189)***
≥ 35 years	32.3	30.8	28.2	27.7	31.2	0.17 (0.111,0.229)***
Education						
No formal school	6.4	11.5	0.0	4.3	0.0	0.04 (−0.078,0.158)
Primary school	61.3	46.2	17.9	8.5	13.1	−0.07 (−0.188,0.048)
Middle school or above	32.3	42.3	82.1	87.2	86.9	0.14 (0.042,0.238)**
No. of births						
1–2	64.5	80.8	69.2	87.2	85.2	0.13 (0.071,0.189)***
> 2	35.5	19.2	30.8	12.8	14.8	0.11 (0.012,0.208)*

*Q* Quintile

*CI* Confidence Interval

*p* < 0.05 *, *p* < 0.01 **, *p* < 0.001 ***

## Discussion

Evidence of inequities in the overall utilization of antenatal care visits was not found, except for at least one antenatal visit in women aged less than 20 years who were rich. We identified inequities in the utilization of delivery by a SBA and cesarean section, particularly in adolescent women and women having the highest education in middle school who were rich. Similar findings of inequities were associated with education level.

No inequity in utilization of ANC found in our study was similar to studies from Thailand and Namibia, even though the wealth indexes were measured by different methods in these studies [9, 34]. This finding in Myanmar could be explained by noting that maternal health is set as a priory public health issue and free essential drugs and services for pregnant women are provided at facilities at the township level [17, 35]. Moreover, our study counted access to ANC as including when a midwife visited a pregnant woman’s home to provide ANC, which is part of the national strategy to ensure adequate services to poor and/or unknowledgeable women [17, 36]. In contrast, inequity in utilization of ANC has been reported in some studies, for example from Malawi and India, with the main reasons of socioeconomic barriers, out-of-pocket payments and illiteracy of the women [37, 38].

We found inequities in the utilization of delivery care both for delivery by a SBA and by cesarean section, which was in accordance with studies from Namibia and rural China which explained the inequities by noting many of the women were poor and uneducated, high hospital fees, and lack of accessibility and availability, which were common in these two countries [34, 39]. A possible explanation for similar inequities in our study might be the limited availability of midwives to provide delivery services, which is different from ANC for which auxiliary midwives are available [26]. In addition, out-of-pocket payments to providers for delivery care was higher than for ANC and limited access to facility-based delivery [14]. A study from Thailand indicated that equity in utilization of delivery by SBA in Thailand was achieved due to expansion of health insurance coverage and a well-functioning primary health care system [9]. For delivery by cesarean section, better access might be due to the fact that women with high socio-economic status have better opportunities to access delivery by cesarean section, which was also found a study from China [39]. Unlike ANC which is provided free of charge, delivery by cesarean section in Uganda and Argentina cost three times more than normal vaginal delivery and poor women could not afford it, which was similar in Myanmar [40].

Although we found no evidence of inequity in the utilization of ANC, the utilization of at least one antenatal visits was more common among women in the richest quintile, particularly those aged less than 20 years. We could not identify the actual reasons, but it could be related to the adolescent in richest quintile had a higher opportunity to seek for knowledge on ANC and use more the service delivery [41–44]. A systematic review published in 2017 confirmed the consistent significance of the utilization of ANC by wealth quintiles in adolescent pregnancy [45].

Likewise, similar findings on inequities in the utilization of delivery by cesarean section could be explained by more opportunity to get a cesarean section among women who are rich and better educated [46, 47]. In general, utilization of cesarean section has been found to be higher in two groups, those with advanced maternal age and younger women who opted for a cesarean section for medical reasons [48]. Some related studies have found that richer and more educated women can access cesarean section more than the poor in some African, Latin American and Southeast Asian countries [49–51].

Positive associations between levels of education and wealth quintiles were revealed in our study. This was not surprising, as rich women generally have higher education along with their higher incomes and they can seek what maternal healthcare they prefer [52]. The findings of inequities related to education rather than wealth were similar. The sample of women who had recently received ANC and delivery care in our study could be regarded as representative of the national situation because the essential characteristics of the women in our study were similar to those described in the results of the Myanmar Demographic and Health Survey 2015 [14]. For determining inequity, we found, as with an earlier study, that accessing data on household assets to create a wealth index was easier and more accurate than accessing data on household income and also provides a relevant measurement for people in low- and middle-income countries [5]. All women responded well to the interview in this study and the response rate was 100%.

There were some limitations of the study. First, this was a cross-sectional study to compare the utilization of ANC and delivery care between poor and rich women using a wealth index; therefore, any potential causal relationship was not definitely determined. Second, the list of women who delivered may have missed some women, but this missing figure, if any, should be small because we retrieved the lists of women who had recently delivered from immunization registers and birth registries, which included women who had and had not delivered by a SBA. Third, the data on utilization of ANC and delivery care were obtained by women’s self-reported experiences, which could have resulted in some recall bias. However, this bias would be minimized because we were only seeking data on the most recent delivery within the previous 12 months. Finally, only the inequity in different subgroups was explored, not testing for the factors associated with the inequities in the utilization of antenatal and delivery care which can be conducted in the future.

## Conclusion

There were no overall inequities in the utilization of ANC, but inequities were substantially found in women who had delivery by cesarean section and/or delivery by a SBA. We suggest that adolescent pregnant women should be the target group to reduce inequity in utilization of delivery care. Appropriate strategies relevant to country context regarding pregnant women in the low wealth index group should be studied to improve the utilization of delivery care.
